# Longer-Term Weight Loss Outcomes Are Not Primarily Driven by Diet Following Roux-en-Y Gastric Bypass and Sleeve Gastrectomy

**DOI:** 10.3390/nu15153323

**Published:** 2023-07-26

**Authors:** Alicia A. Sorgen, Anthony A. Fodor, Kristine J. Steffen, Ian M. Carroll, Dale S. Bond, Ross Crosby, Leslie J. Heinberg

**Affiliations:** 1 Department of Bioinformatics and Genomics, University of North Carolina, Charlotte, NC 28223, USA; asorgen@uncc.edu (A.A.S.); afodor@uncc.edu (A.A.F.); 2 Department of Pharmaceutical Sciences, College of Health Professions, North Dakota State University, Fargo, ND 58103, USA; kristine.steffen@ndsu.edu; 3 Sanford Center for Biobehavioral Research, Fargo, ND 58122, USA; ross.crosby@sanfordhealth.org; 4 Department of Nutrition, Gillings School of Global Public Health, University of North Carolina, Chapel Hill, NC 27599, USA; ian_carroll@med.unc.edu; 5 Departments of Surgery and Research, Hartford Hospital/Hartford Healthcare, Hartford, CT 06106, USA; dale.bond@hhchealth.org; 6 Department of Psychiatry and Psychology, Cleveland Clinic Lerner College of Medicine, Cleveland, OH 44195, USA

**Keywords:** dietary intake, bariatric surgery, weight loss, outcomes, macronutrient consumption

## Abstract

Metabolic and bariatric surgery (MBS) is the most effective long-term treatment for Class III obesity. Reduced dietary intake is considered a behavioral driver of post-surgical weight loss, but limited data have examined this association. Therefore, this study examined prospective, longitudinal relationships between dietary intake and weight loss over 24 months following Roux-en-Y Gastric Bypass and Sleeve Gastrectomy. Relationships between weight loss and dietary intake were examined using a validated 24-h dietary recall method. Associations between total energy/macronutrient intake and weight loss outcomes were assessed at 12-, 18-, and 24-months following MBS, defining patients as “responders” and “suboptimal responders”. Consistent with previous literature, 12-month responders and suboptimal responders showed significant associations between weight loss and energy (*p* = 0.018), protein (*p* = 0.002), and total fat intake (*p* = 0.005). However, this study also revealed that many of these associations are no longer significant 24 months post-MBS (*p* > 0.05), despite consistent weight loss trends. This study suggests a short-term signal between these dietary factors and weight loss outcomes 12 months post-MBS; however, this signal does not persist beyond 12 months. These results are essential for interpreting and designing clinical studies measuring long-term post-surgical weight loss outcomes.

## 1. Introduction

Metabolic and bariatric surgery (MBS) is the most effective treatment for severe obesity [1], with sleeve gastrectomy (SG) and Roux-en-Y gastric bypass (RYGB) being the most commonly performed procedures [2,3]. Although definitions of “success” vary, a standard convention is to define an optimal outcome of surgery as one in which a patient loses at least 50% of their excess weight, with less-than-optimal outcomes occurring when excess weight loss (EWL) is less than 50% [4,5]. A relatively large body of literature has examined predictors of lower-than-expected weight loss following surgery. Predictors have included a lack of sustained nutritional management and physical activity coupled with problematic post-surgical eating behaviors [6,7]. The mechanisms underlying MBS weight loss outcomes vary; however, dietary intake after surgery has been shown to play a crucial role in weight loss and weight loss maintenance [8,9]. Decreasing energy consumption while increasing energy expenditure is considered a foundational aspect of weight loss, as recommended by the World Health Organization [10], the Centers for Disease Control [11], and the National Institutes of Health [12]. Patients generally receive nutritional education before and after surgery and are provided with specific guidelines to maintain sufficient nutrient intake [9]. Such post-operative guidelines include a recommended energy intake from carbohydrates of 35–48% with at least 60–80 g of protein per day [13]. Some research has shown reduced energy intake to be a main behavioral driver of initial weight loss [14,15]. However, previous studies have not sufficiently controlled for dietary intake, reflecting a deficit in the current literature [16].

Although MBS is the most effective treatment for Class III obesity, several studies, including the multi-site Longitudinal Assessment of Bariatric Surgery (LABS), have found that a substantial portion of patients (~25%) do not achieve their expected weight loss outcomes and that most patients regain some of their lost weight [17,18]. Although longer-term weight loss outcome studies have been conducted, very few have investigated dietary intake and weight loss outcomes over the past two years post-MBS.

A retrospective single-site 2020 study conducted by Lim et al. in Seoul, Korea, examined energy and nutrient intake changes in patients up to 12 months post-MBS [19]. The study classified patients into two groups depending on whether they lost 50% of their excess weight 12 months after MBS. The results revealed that patients who lost over half of their excess weight had significantly lower daily total caloric, carbohydrate, and fat intake than the sub-optimal group and significantly higher protein intake [19]. Lim et al. also attempted to define caloric and macronutrient daily intake cutoffs as recommendations for achieving optimal weight loss outcomes, emphasizing that 12-month post-MBS weight loss is related to the patients’ compliance with these recommendations.

The present study sought to reproduce these previous findings in a different setting and extend them to 18- and 24-months post-surgery, as studies have shown the majority of weight loss to occur within the first year [18]. Due to the prevalence of weight recurrence after one year, it is likely that the associations between energy/macronutrient consumption and weight loss are weakened or no longer observed with long-term outcomes. This study was conducted with a starting cohort of 145 MBS participants from Cleveland, OH, and Fargo, ND, to further aid in interpreting and designing long-term clinical studies on weight loss and dietary intake.

## 2. Materials and Methods

### 2.1. Participants and Recruitment

Participant recruitment took place through the Sanford Center for Biobehavioral Health (Fargo, ND) and the Cleveland Clinic (Cleveland, OH) and was approved by the Institutional Review Boards at both sites. This NIH-funded study (1RO1 DK112585-01 and 3R01DK112585-01) is registered on ClinicalTrials.gov (Trial ID NCT03065426), and the protocol for the study has been described previously [20].

Participants in the pre-operative evaluation process for RYGB and SG were offered the opportunity to volunteer for this study. Enrollment criteria (detailed in Table S1) were carefully selected to balance generalizability with the need to limit confounding variables. A total of 145 participants undergoing bariatric surgery were initially enrolled in this study; however, 124 proceeded to surgery and were in the analyzable sample.

### 2.2. Study Design and Timeline

This study was designed as part of a larger, prospective longitudinal assessment of behavioral, biological, and physiological influences related to weight loss over a two-year study period following RYGB or SG. Participants provided informed consent during a screening visit at the research center. They completed a medical history assessment for study eligibility according to the inclusion and exclusion criteria list [20]. Eligible participants then completed comprehensive dietary diaries and were measured for relevant biometrics just before their respective surgeries (baseline) and at specified follow-up time points (1, 6, 12, 18, and 24 months) post-surgery.

### 2.3. Biometrics and Dietary Recall

Height (in) and weight (lb) were recorded at each study visit, with participants wearing lightweight clothing without shoes. Measurements were converted to metric units (m and kg, respectively), and the body mass index (BMI) was calculated. An individual’s ideal body weight (IBW) was established based on an “ideal” BMI of 25 kg/m^2^. Excess weight was calculated by subtracting IBW from the recorded weight, and the percentage of excess body weight loss (%EWL) was determined by dividing the difference between actual weight loss and “ideal” weight loss (Equation (1)).

(1) $$\%\mathrm{EWL}=\left(\frac{{\mathrm{Weight}}_{\mathrm{preop}}-{\;\mathrm{Weight}}_{\mathrm{postop}}}{{\mathrm{Weight}}_{\mathrm{preop}}-{\;\mathrm{Weight}}_{\mathrm{ideal}}}\right)\;\times \;100$$

Participants were classified as “responders” or “suboptimal responders” based on their %EWL at 12, 18, and 24 months. Per widely utilized clinical standards and for direct comparison with previous findings, those exhibiting > 50% excess weight loss at a given time point were considered “responders”, and those with an excess loss of <50% were considered “suboptimal responders” [5].

Nutritional intake data were collected using the Automated Self-administered 24-h (ASA24) Dietary Assessment Tool, a 24-h dietary recall developed by the National Cancer Institute [21]. The ASA24 is a validated, widely used method for assessing dietary intake, including foods, fluids, vitamins, and supplements [22]. Participants completed three days of the ASA24 dietary recall at each time point (with the goal of including at least one weekday and one weekend day, though this goal was met ~68% of the time throughout the study) to estimate total mean energy (kilocalories) and macronutrient (grams) consumption.

### 2.4. Statistical Analysis

Participants were classified as responders and suboptimal responders based on %EWL data at 12 months (12-month outcome group), 18 months (18-month outcome group), and 24 months (24-month outcome group) post-surgery. Due to individual time-based differences, participants could be classified as responders at one time point but as suboptimal responders at another, resulting in different sub-sample sizes. At each time point, responder and suboptimal responder groups were compared for differences in weight by surgery type and nutritional intake using Wilcoxon’s rank-sum test. Associations between %EWL and diet (i.e., daily total kilocalories and grams of protein, carbohydrates, and fats) at 12-, 18-, and 24-months post-surgery were assessed with non-parametric Spearman rank correlations. Univariate linear regression analyses were implemented for longitudinal factors affecting excess weight loss outcomes using R’s “lm” function [23]. All statistical analyses were performed using the automated BioLockJ pipeline (https://github.com/BioLockJ-Dev-Team/BioLockJ, accessed on 16 December 2021), utilizing a Dockerized version of R (version 4.0.2) to ensure study reproducibility.

## 3. Results

### 3.1. Patient Characteristics

A total of 145 participants were recruited, though 19 of the initial patients did not proceed to surgery, and two RYGB patients were lost to follow-up, resulting in 124 participants (89 RYGB and 37 SG) for analysis (Figure S1). The number of patients recruited from each study location was roughly equal (48.4% from Fargo and 51.6% from Cleveland), with no significant variation in baseline BMI among patients at either location (*p* = 0.132). Of the 124 participants who underwent surgery and attended at least one follow-up visit, 93 (75%) had complete data for this analysis at the 24-month time point. The average age of participants was 42.8 years, including patients of Caucasian (74.2%), Black or African American (18.5%), Hispanic or Latino (1.6%), Native Hawaiian/Pacific Island (0.8%), or more than one race/ethnicity (4.8%), and 80.6% were female (Table S2). These demographics are consistent with national bariatric surgery statistics [24]. There was no significant differentiation in baseline BMI between RYGB (44.28 ± 5.66 kg/m^2^) and SG (47.46 ± 8.82 kg/m^2^) patients prior to surgery (*p* = 0.348).

### 3.2. Excess Weight Loss and Responder Status

**Figure 1:**
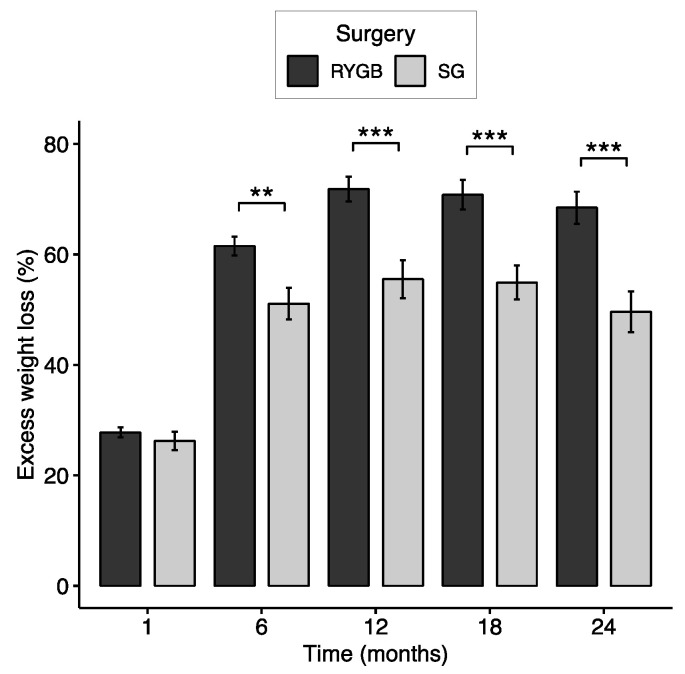
Excess weight loss in RYGB and SG patients at 1, 6, 12, 18, and 24 months. Data are presented as means ± standard errors. Pairwise statistical differences are analyzed by Wilcoxon’s rank-sum test. ** indicates *p* < 0.01, and *** indicates *p* < 0.001 RYGB versus SG.

The mean pre-operative BMI of the 124 patients who proceeded to surgery was 45.93 ± 6.79 kg/m^2^, with an average of 60.1 ± 21.17 kg of excess weight based upon a BMI of 25 kg/m^2^ (Table 1). The average %EWL at one-, six-, 12-, 18-, and 24-months post-operation were 27.3%, 58.3%, 66.5%, 65.5%, and 62.6%, respectively (Table 1). The percentage of excess weight carried by RYGB and SG patients did not differ before surgery (*p* = 0.348) or within the first post-surgical month (*p* = 0.485). By six months, RYGB patients exhibited significantly more excess weight loss than SG patients, and this difference remained consistent throughout the duration of the study (Figure 1, Table 1).

At 12 months, out of the remaining 110 participants, 85 (77.3%) were classified as “responders,” with more RYGB recipients classified as responders (86.5%) than SG recipients (58.3%). By 18 months, 77.2% of the participants were in the responder category, compared with 68.8% by month 24. Again, differences by procedure persisted, with a higher percentage of RYGB participants being in the responder category compared with SG at 18 (88.1% of RYGB and 55.9% of SG) and 24 (82.8% of RYGB and 37.9% of SG) months, respectively. Excess weight loss from baseline was highly correlated (*p* < 0.001) across the 12-, 18-, and 24-month timepoints (Figure S2). Irrespective of surgery type, individual patient weight loss trends after 12 months post-surgery remained highly stable, with most patients maintaining the same optimal responder and suboptimal responder status (Figure S2, red and orange symbols). However, a few patients moved between responder and suboptimal responder status at the 18- and 24-month time points (Figure S2b,c, blue and green symbols).

### 3.3. Changes in Total Energy and Macronutrient Intake

**Figure 2:**
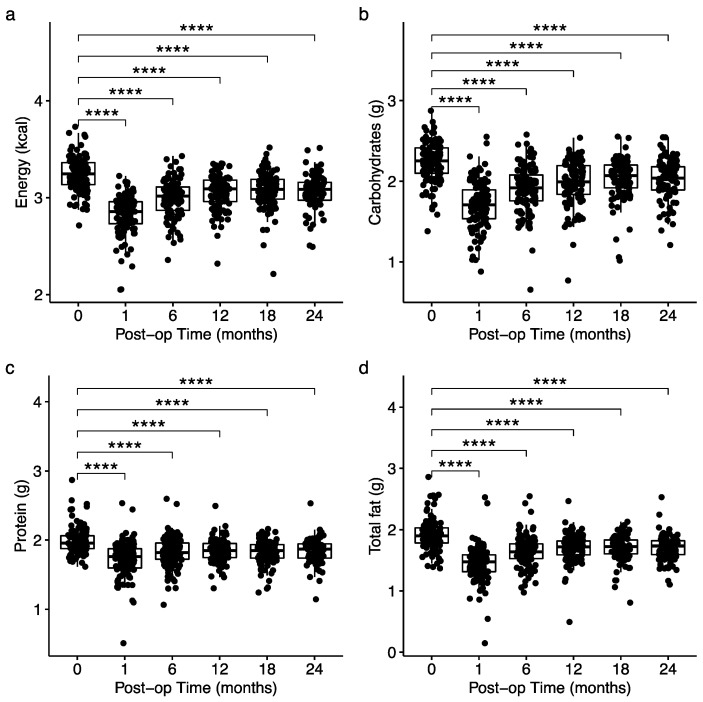
Differences in dietary intake for all patients over time for total energy (**a**), carbohydrates (**b**), protein (**c**), and fat (**d**). Pairwise statistical differences are analyzed by univariate linear regression. **** indicates *p* < 0.0001 baseline versus post-surgery time.

Energy and macronutrient intake significantly decreased after surgery (Figure 2). Differences in early post-surgical protein (g) and total fat (g) intake were observed between RYGB and SG patients. SG patients showed higher total protein intake (g) at one and six months and higher total fat intake (g) at one month relative to RYGB patients (Table S3). No differences were found in energy (kcal) or total carbohydrate (g) intake between surgical types at any assessment point, and no differences in dietary intake were observed between surgery types after six months (Table S3).

### 3.4. Direct Associations between Nutrient Intake and %EWL at 12-, 18-, and 24-Months

**Figure 3:**
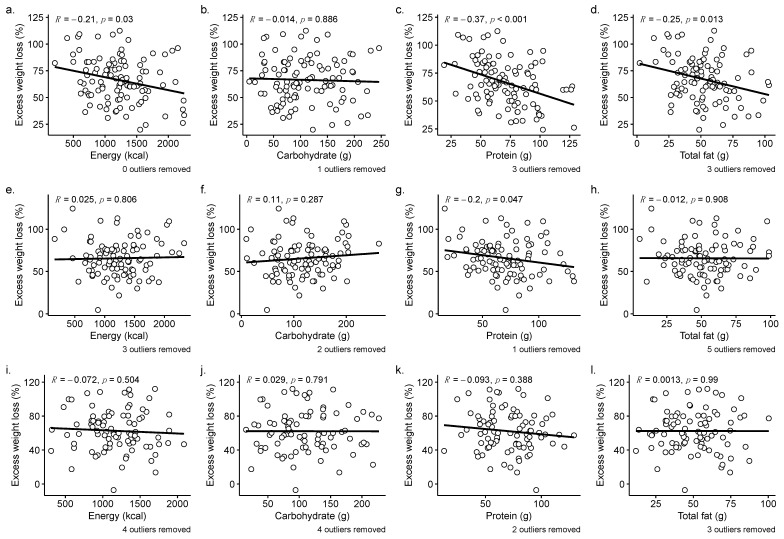
Correlations between excess weight loss and dietary intake at 12 months (**a**–**d**), 18 months (**e**–**h**), and 24 months (**i**–**l**) post-surgery with outliers removed. The number of outliers removed from the analysis is indicated below each plot. Correlation coefficients (*r*) and *p*-values were calculated using Spearman’s rank correlation. Grey shading indicates a confidence interval of 95%.

Direct relationships between dietary variables and excess weight loss at different time points revealed that daily energy intake (kcal) significantly negatively correlated with weight loss (%EWL) at 12 months (*p* = 0.030, *r* = −0.21), but this association was not significant at the 18- (*p* = 0.821) and 24-month (*p* = 0.504) time periods (Figure 3a,e,i). Likewise, total fat intake showed a negative association with %EWL at 12 months post-surgery (*p* = 0.013, *r* = −0.29; Figure 3d). However, this association was also no longer significant after 12 months (Figure 3h,l). Protein intake showed a significant negative correlation to %EWL at 12 months (*p* < 0.001, *r* = −0.37; Figure 3c) and at 18 months (*p* = 0.026, *r* = −0.22; Figure 3g), but at 24-months, this association was no longer significant (*p* = 0.388; Figure 3k). Patient weight loss showed no correlation with carbohydrate intake at 12, 18, or 24 months (Figure 3b,f,j). These findings are observed with and without outliers, indicating a few extreme data points did not drive these associations.

### 3.5. Changes in Total Energy and Macronutrient Intake over Time by Weight Loss Response

Given that associations between diet and %EWL seen at 12 months did not extend to later time points (Figure 3), the present study sought to expand upon a previous study on patient weight loss outcomes (determined with a 50% EWL threshold) and diet with a 12-month study termination [19]. The present analysis considered how diet and weight loss associations would be affected by study duration length (12, 18, or 24 months), with patients assigned responder or suboptimal responder status according to the same 50% EWL threshold at each designated study endpoint.

#### 3.5.1. Dietary Intake and Weight Loss Outcomes at 12 Months

Pre-surgery nutritional intake did not differ among patients classified as responders (*n* = 85) and suboptimal responders (*n* = 25) at the 12-month study visit (Table 2). The 12-month responders consumed, on average, 669 kcal one month after surgery, while suboptimal responder caloric intake was significantly higher by ~260 kcal (*p* < 0.001). These suboptimal responders continued to consume significantly more calories than responders at six and 12 months (Table 2). Carbohydrate intake differed between the outcome groups at one- and six-months post-surgery, but this difference did not persist at 12 months (Table 2). At 12 months, the protein intake of suboptimal responders was significantly higher than the responder intake (*p* = 0.002). However, no differences were observed between groups in prior months (Table 2). 12-month suboptimal responders were found to have significantly higher total fat intake than responders after surgery (Table 2). The carbohydrate energy ratio was found to be significantly higher among the responder group at 12 months (*p* = 0.025), but no other differences in macronutrient energy ratios were found among these outcome groups (Figure S3).

#### 3.5.2. Dietary Intake and Weight Loss Outcomes at 18 Months

When classifying patients at an 18-month termination point, 76 were deemed responders and 22 were suboptimal responders (Table S4). 18-month responders and suboptimal responders again showed no differences in dietary intake prior to surgery. However, after surgery, protein intake was consistently higher in the suboptimal responder group (1 month p = 0.029; 6 month p = 0.014; 12 month p = 0.002; 18 month p = 0.003). Energy intake was significantly increased among suboptimal responders shortly after surgery, at one (p = 0.003) and six months (p = 0.021), but did not significantly vary from responder intake after 12 months. Suboptimal responders consumed significantly more total fat than the responder group six months post-surgery (p = 0.022). No differences were observed in carbohydrate intake at any time between these outcome groups. These results remain consistent with the 12-month observations in this study.

#### 3.5.3. Dietary Intake and Weight Loss Outcomes at 24 Months

At the 24-month post-surgery, there was a ~10% decrease in the number of responders (Figure S2), with 62 responders and 28 suboptimal responders yielding a ~69% response rate. Although this difference in response rate was modest, it had significant effects on the analyses in that the associations between %EWL and nutrient intake no longer reflected those found with the 12-month outcome groups. The 24-month outcome groups again showed no significant differences in protein, carbohydrate, or caloric intake pre-operatively; however, most dietary intake variability between the responders and suboptimal responders was no longer significant, and no differences were observed in carbohydrate intake between these outcome groups at any time (Table 3). Total fat intake was found to be significantly higher among pre-surgery suboptimal responders (p = 0.048), although no differences in fat intake were observed between groups post-surgery. 24-month responders consumed significantly fewer calories in one month post-surgery (p = 0.011) but were not significantly different from their suboptimal counterparts at later times. Only protein intake was found to differ between outcome groups at later time points (12 and 18 months), but by the 24-month study’s end, this difference was no longer observed. These results are inconsistent with the short-term (12- and 18-month) diet and weight loss associations observed in the present study.

### 3.6. Differences in Sample Size and Power Do Not Explain Differences in Outcome between 12 and 24 Months

To determine if sample size was the main contributor to the differences in weight loss and diet associations at 12 and 24 months, a subset of the final 93 patients present in the study at 24 months were classified according to their 12-month outcome status, effectively reducing the 12-month analysis sample size. With the limited sample size (n = 90; three patients who provided 24-month data were unable to report for their 12-month follow-up), the associations between weight loss and diet were comparable to those found in the complete data set (Table S5). Responders (n = 69) consumed significantly fewer calories than suboptimal responders (n = 21) at one (*p* < 0.001) and six months (*p* = 0.01), though a significant difference was no longer observed at 12 months (*p* = 0. 064). All comparisons between groups for carbohydrates, protein, and total fat remained consistent with the results found in the full 12-month dataset, indicating that sample size is not driving the association changes.

## 4. Discussion

To further elucidate the role of dietary intake in post-bariatric surgery weight loss outcomes, the present study sought to examine associations between short- and long-term total energy and macronutrient intake and patient weight loss outcomes. This study aimed to test whether relationships between post-MBS dietary restrictions and weight loss are maintained long-term past the first year of surgery.

A previous study conducted in South Korea by Lim et al. (2020) found no significant differences in pre-surgical diet between outcome groups but did observe significantly lower fat, carbohydrate, and caloric intake among their responder group (*n* = 127) at 6 and 12 months, suggesting an association between a patient’s post-surgical dietary intake and their weight loss outcome after one year. The present study expanded upon the previous analysis using the same weight loss outcome threshold, dietary measures, and statistical methods on a cohort of 124 BS patients from the U.S., with data collection extending to 24 months post-surgery. The results were largely consistent among the two study cohorts, with weight loss outcomes measured at 12 months. No differences in dietary intake were observed prior to surgery between 12-month outcome groups, suggesting there was no underlying variability in patient diets before surgical intervention. The responder group showed significantly different intake volumes of protein, total fat, and total calories than their suboptimal counterparts at 12 months post-surgery.

However, significant diet and weight loss associations were no longer observed with an extended study duration of 24 months (Table 3). Similarly, total energy, protein, and fat intake significantly correlate with excess weight loss at the 12-month but not 24-month time point. These results strongly suggest that dietary variables may be associated with patient outcomes in the first 12 months post-surgery but that these associations are no longer significant at 24 months.

The differences in dietary intake among outcome groups at 12 months were no longer observed at 24 months but may be explained by a relatively small number of patients moving from responder to suboptimal responder status (Figure S2). Since defining responders and suboptimal responders is a somewhat arbitrary metric, direct relationships between %EWL and macronutrients were examined (Figure 3); and, again, the demonstrated associations at the 12-month time point were no longer observed at 24 months, highlighting that the early associations with dietary factors become less robust in the second year following surgery and that other factors may explain different weight outcomes.

The loss of associations between patient outcomes and diet could not be attributed to the reduced sample size at 24 months. Analysis of 12-month outcomes and dietary factors using the same reduced sample size still yielded the same associations observed with the full 12-month dataset, indicating that the loss of these associations is not simply due to a lack of statistical power in the smaller 24-month dataset (Table S5). While statistically significant and reproducible with the Lim et al. study, the amount of weight loss variability that dietary factors explain in this study is small (R^2^ < 0.15; Table 2).

Numerous studies have addressed various factors associated with weight loss after bariatric surgery; however, most have been limited by small sample sizes and short follow-up durations, typically 12 months [25]. Consistent with previous literature, this study found that patients lost significantly more weight with RYGB than with SG (Figure 1). However, this study suggests that dietary variables do not play a major role in weight loss outcomes between the two surgery types. Despite higher protein and fat intake among SG patients in the early post-surgical months, RYGB and SG patients showed little difference in dietary intake throughout the study (Table S3).

Additionally, patient’s weight loss was found to stabilize after the first 12 months of surgery, with minimal difference in 12- to 24-month weight change regardless of surgery type or outcome consistency (Figure S2). Dietary intake, however, showed much more variability (Figure S4). Though energy intake may be important following surgery in the short term, several putative mechanisms of action have been offered that explain alternate outcomes beyond energy balance [26]. The variability of longer-term (beyond the first year) weight loss outcomes after surgery is well documented and can depend on numerous factors, including the age of the patient undergoing surgery [27]; the initial surgery type and subsequent surgical revisions or reversals [28]; sedentary behavior and physical activity [1,29]; the incidence and remission of diabetes and other comorbid conditions [28,30]; disordered eating patterns, including binge eating and loss of control, developed before and after surgery [27,29,31]; and other behaviors associated with negative health (smoking and alcohol consumption) [30]. Psycho-behavioral factors such as mood and executive function [32] and biological factors, including the intestinal microbiome [25,33], can also have an influence on patient weight loss. Additionally, dietary underreporting has been associated with higher BMI, making it possible that patients with suboptimal outcomes are underestimating their intake [27]. Weight regains may also be a factor in patient follow-up reporting that can affect long-term outcome results [34].

After MBS, patients are at risk of protein malnutrition and, as a result, are informed by their healthcare provider that they need approximately 1.1–1.5 g of protein per kg of ideal body weight per day, with 10–35% of their total energy intake being derived from a protein source [8]. In terms of net intake (g) and energy ratios (%), patients from this study cohort were largely non-adherent to these guidelines and recommendations, regardless of weight loss outcomes (Figure S5). Analysis of macronutrient energy ratios over time reveals few changes in the quality of patient diets, except for significant reductions in carbohydrate- and fat-derived energy in the post-surgery diet of suboptimal responders classified at 24 months (Figure S6). These findings indicate that patients are not eating higher-quality diets but simply consuming fewer calories, suggesting that other factors (e.g., the gut microbiome) are at play in predicting patient weight loss outcomes [35].

Classifying the outcome of bariatric surgery by %EWL and further denoting the success of surgery according to the 50% mark as defined in 1982 may not be the optimal strategy for defining longer-term weight outcomes following surgery [4]. The goal of bariatric surgery is to improve health-related quality of life and reduce health risks, which cannot be captured by a %EWL threshold, particularly in cases of metabolic surgeries like RYGB [5]. Further, the 50% EWL Reinhold threshold was characterized before the development and implementation of the widely performed sleeve gastrectomy and does not account for factors associated with this procedure [36]. Despite these limitations, the present study utilized the Reinhold criteria of 50% excess weight loss to directly compare and advance previous research.

Though improvements in dietary tracking have been made, patient-reported dietary recall methods can still be unreliable, and misreported data could have influenced these results [37,38]. For example, the ASA24 dietary recall method utilized in this study has been found to underestimate protein intake when measured by 3-day intake averages [38]. More accurate intake measurements could be collected with an increase in the number of recall days and a decrease in the amount of time between each recall assessment [39], but these increase participant burden, which is a significant factor in longitudinal studies with repeat assessments. Future studies may be able to utilize improvements in dietary recall accuracy and may therefore show stronger associations with weight loss than the small but significant associations shown here.

This study was able to reproduce the short-term dietary associations observed by Lim et al. despite being performed in the U.S., while the Lim et al. study was conducted in South Korea. This reproducibility is notable as dietary behaviors widely vary based on geography and culture [40,41]. Though the sample size was less robust than the previous study, analyzing a subset of patients at 12 months indicated that sample size was not the cause of the diminished associations found at later time points, though more sample data might certainly increase the power. The current analysis was strengthened as a prospective, dual-site study with an extended duration. Patient retention was calculated at 92% by one-year follow-up and 75% by two years. Though the final cohort was slightly less than the ideal 80% original cohort follow-up rate [34], the retention rate in this prospective study decreases the risk of potential bias associated with patient dropout. These factors aided in advancing the short-term research on post-MBS dietary intake and weight loss with the use of a rigorous dietary method and follow-up out to 24 months. The results suggest the intriguing possibility that patients’ weight may not be closely associated with dietary intake after 12 months post-surgery, contrary to conventional thought. This study shows the importance of designing clinical studies with sampling beyond 12 months to properly assess the impact of dietary factors on long-term patient success. Future long-term studies will be needed to continue to elucidate patient attributes that have the most considerable effect on weight loss and regain over multi-year durations.

## Figures and Tables

**Table 1:** Patient weight metrics.

Timepoint	*n*	Weight (kg)	BMI (kg/m^2^)	Percent Loss (%)	EWL (%)
Baseline	**124**	**131.64 ± 25.21**	**45.93 ± 6.79**	-	-
	RYGB—87	129.51 ± 21.58	45.28 ± 5.66	-	-
	SG—37	136.66 ± 31.97	47.46 ± 8.82	-	-
Postop 1 month	**122**	**115.89 ± 22.41**	**40.52 ± 6.37**	**11.81 ± 3.44**	**27.32 ± 8.94**
	RYGB—86	114.07 ± 18.46	39.9 ± 5.02	12.02 ± 3.4	27.77 ± 8.49
	SG—36	120.23 ± 29.68	41.99 ± 8.72	11.31 ± 3.55	26.23 ± 9.96
Postop 6 months	**114**	**97.85 ± 20.93**	**34.37 ± 6.09**	**25.3 ± 5.95**	**58.32 ± 16.36**
	RYGB—79	94.29 ± 15.76	33.26 ± 4.58	26.69 ± 5.66 ***	61.53 ± 15.16 **
	SG—35	105.88 ± 28.12 *	36.88 ± 8.1 *	22.15 ± 5.43	51.09 ± 16.88
Postop 12 months	**110**	**93.13 ± 21.66**	**32.69 ± 6.34**	**28.79 ± 7.99**	**66.51 ± 21.19**
	RYGB—74	88.32 ± 16.32	31.12 ± 4.68	31 ± 7.29 ***	71.85 ± 19.44 ***
	SG—36	103.02 ± 27.5 **	35.91 ± 7.99 **	24.23 ± 7.52	55.53 ± 20.64
Postop 18 months	**101**	**93.31 ± 21.76**	**32.73 ± 6.16**	**28.25 ± 8.48**	**65.46 ± 21.93**
	RYGB—67	88.61 ± 17.32	31.21 ± 4.86	30.46 ± 8.57 ***	70.81 ± 21.98 ***
	SG—34	102.58 ± 26.5 **	35.73 ± 7.32 **	23.89 ± 6.45	54.93 ± 17.86
Postop 24 months	**93**	**94.87 ± 21.64**	**33.36 ± 6.41**	**27.25 ± 9.26**	**62.59 ± 23.8**
	RYGB—64	89.3 ± 17.45	31.62 ± 5.03	29.45 ± 9.01 ***	68.47 ± 23.24 ***
	SG—29	107.18 ± 25 **	37.19 ± 7.5 **	22.42 ± 7.99	49.62 ± 19.82

Data are reported as mean ± SD. Data in bold represent values for all patients at each timepoint with values per surgery type below. *p*-values were computed by Wilcoxon’s rank-sum test for RYGB versus SG weight metrics and indicated where sample means are greater; * indicates *p* < 0.05, ** indicates *p* < 0.01, *** indicates *p* < 0.001.

**Table 2:** Comparison of nutrition intake between groups for patients characterized as responders vs. suboptimal responders at 12 months post-surgery.

		Responder	Suboptimal Responder		
		(*n* = 85)	(*n* = 25)	R^2^	*p*-Value
Baseline	Weight (kg)	126.01 ± 21.13	146.67 ± 31.57	0.118	**0.002**
	Energy (kcal)	1865.58 ± 842.97	1973.37 ± 756.42	0.003	0.392
	Carbohydrates (g)	198.29 ± 119.14 (43.7%)	209.78 ± 101.33 (46.5%)	0.002	0.448
	Protein (g)	109.6 ± 86.56 (27.7%)	117.28 ± 70.75 (30.2%)	0.002	0.267
	Total fat (g)	98.8 ± 94.24 (52.4%)	111.46 ± 70.2 (62.8%)	0.004	0.053
Postop	Weight (kg)	110.27 ± 18.12	130.76 ± 29.13	0.143	**0.001**
1 month	Energy (kcal)	668.69 ± 253.94	930.85 ± 295.61	0.148	**<0.001**
	Carbohydrates (g)	58.41 ± 50.72 (35.8%)	79.96 ± 47.85 (33.5%)	0.031	**0.013**
	Protein (g)	59.97 ± 45.52 (36.8%)	64.17 ± 22.47 (28.9%)	0.002	0.105 ^Δ^
	Total fat (g)	33.71 ± 44.01 (46.7%)	39.33 ± 17.15 (37.5%)	0.003	**0.007**
Postop	Weight (kg)	91.35 ± 15.01	119.82 ± 25.38	0.311	**<0.001**
6 months	Energy (kcal)	1021.7 ± 427.11	1285.95 ± 458.12	0.06	**0.009** **^Δ^**
	Carbohydrates (g)	91.51 ± 63.07 (35.1%)	110.9 ± 54.65 (34.4%)	0.017	**0.038** **^Δ^**
	Protein (g)	74.4 ± 53.24 (30.9%)	76.09 ± 28.62 (24.0%)	<0.001	0.247
	Total fat (g)	50.88 ± 48.29 (45.0%)	59.21 ± 25.22 (41.2%)	0.006	**0.017** **^Δ^**
Postop	Weight (kg)	85.72 ± 14.63	118.35 ± 22.9	0.402	**<0.001**
12 months	Energy (kcal)	1156.08 ± 416.19	1436.55 ± 466.82	0.072	**0.018** **^Δ^**
	Carbohydrates (g)	110.25 ± 61.81 (36.8%)	116.61 ± 58.31 (31.1%)	0.002	0.685 ^Δ^
	Protein (g)	71.27 ± 36.12 (26.0%)	85.48 ± 24.49 (25.1%)	0.031	**0.002** **^Δ^**
	Total fat (g)	52.42 ± 33.14 (40.6%)	66.43 ± 25.31 (41.5%)	0.034	**0.005** **^Δ^**

Data are reported as mean ± SD. *p*-values were computed by Wilcoxon’s rank-sum test. The data in parentheses report the average energy ratio for each macronutrient intake. R^2^ was calculated by univariate linear models. Bold *p*-values indicate a *p* < 0.05 responder versus a suboptimal responder. ^Δ^ corresponds with the significant comparisons observed by Lim et al. [19].

**Table 3:** Comparison of nutrition intake between groups for patients characterized as responders vs. suboptimal responders at 24 months post-surgery.

		Responder	Suboptimal Responder		
		(*n* = 64)	(*n* = 29)	R^2^	*p*-Value
Baseline	Weight (kg)	126.81 ± 19.42	138.62 ± 32.7	0.049	0.121
	Energy (kcal)	1807.96 ± 681.19	1820.37 ± 762.13	<0.001	0.879
	Carbohydrates (g)	179.03 ± 81.77 (38.7%)	222.75 ± 161.77 (53%)	0.032	0.673
	Protein (g)	91.19 ± 28.57 (21.8%)	141.41 ± 141 (38.3%)	0.077	0.167
	Total fat (g)	76.24 ± 31.16 (37.7%)	133.3 ± 142.57 (79.2%)	0.094	**0.048**
Postop	Weight (kg)	110.99 ± 16.18	124.25 ± 30.1	0.077	**0.042**
1 month	Energy (kcal)	676.96 ± 263.61	833.98 ± 291.5	0.068	**0.011**
	Carbohydrates (g)	58.46 ± 49.11 (34.8%)	69.71 ± 41.96 (32.8%)	0.012	0.094
	Protein (g)	58.87 ± 42.55 (36.9%)	62.09 ± 24.22 (30.4%)	0.002	0.229 ^Δ^
	Total fat (g)	32.14 ± 41.13 (45%)	34.11 ± 15.19 (36.6%)	0.001	0.091
Postop	Weight (kg)	92 ± 14.54	110.37 ± 27.77	0.162	**<0.001**
6 months	Energy (kcal)	1016.07 ± 434.96	1206.94 ± 488.45	0.037	0.053 ^Δ^
	Carbohydrates (g)	90.74 ± 67.43 (35.1%)	99.74 ± 53.14 (33%)	0.004	0.205 ^Δ^
	Protein (g)	77.92 ± 60.31 (32.5%)	75.54 ± 31.86 (25.5%)	<0.001	0.39
	Total fat (g)	53.3 ± 55.04 (47.7%)	54.91 ± 26.24 (40.2%)	<0.001	0.086 ^Δ^
Postop	Weight (kg)	85.46 ± 14.82	109.01 ± 26.16	0.252	**<0.001**
12 months	Energy (kcal)	1143.68 ± 426.09	1338 ± 476.48	0.04	0.141 ^Δ^
	Carbohydrates (g)	108.7 ± 66.45 (36.3%)	109.96 ± 57.76 (31.6%)	<0.001	0.905 ^Δ^
	Protein (g)	72.11 ± 38.97 (26.9%)	84.65 ± 25.65 (26.9%)	0.026	**0.005** **^Δ^**
	Total fat (g)	52.15 ± 36.69 (40.8%)	61.4 ± 27.24 (40.8%)	0.016	0.109 ^Δ^
Postop	Weight (kg)	85.99 ± 14.88	110.35 ± 24.31	0.28	**<0.001**
18 months	Energy (kcal)	1261.91 ± 498.21	1364.14 ± 530.58	0.009	0.467
	Carbohydrates (g)	121.91 ± 61.32 (37.4%)	121.67 ± 60.63 (35.3%)	<0.001	0.754
	Protein (g)	65.56 ± 21.83 (22.7%)	84.3 ± 28.94 (25.2%)	0.115	**0.003**
	Total fat (g)	53.67 ± 23.26 (38.3%)	59.44 ± 25.43 (39.1%)	0.012	0.402
Postop	Weight (kg)	86.36 ± 14.77	113.67 ± 22.7	0.346	**<0.001**
24 months	Energy (kcal)	1218.42 ± 472.41	1296.49 ± 542.18	0.005	0.451
	Carbohydrates (g)	116.14 ± 62.55 (39.9%)	121.33 ± 71.11 (36.3%)	0.001	0.808
	Protein (g)	75.65 ± 41.86 (28.6%)	71.23 ± 22.48 (23.9%)	0.003	0.976
	Total fat (g)	56.64 ± 43.52 (47.6%)	53.28 ± 21.87 (38%)	0.002	0.724

Data are reported as mean ± SD. *p*-values were computed by Wilcoxon’s rank-sum test. The data in parentheses report the average energy ratio for each macronutrient intake. R^2^ was calculated by univariate linear models. Bold *p*-values indicate a *p* < 0.05 responder versus a suboptimal responder. ^Δ^ corresponds with the significant comparisons observed by Lim et al. [19].

## Data Availability

Data will be available to the extent the NIH requires and is permitted by appropriate laws and rules. It will be available after the study’s conclusion and when the data has been cleaned and the patient data is fully de-identified.

## Supplementary Materials

The following supporting information can be downloaded at: https://www.mdpi.com/article/10.3390/nu15153323/s1. Table S1. Study enrollment criteria; Figure S1. Infographic for participant attrition; Table S2. Baseline demographics for all participants; Figure S2. Patient excess weight loss (%) from baseline weight; Table S3. Differences in dietary intake between RYGB and SG patients; Figure S3. Energy ratio comparisons between outcome groups; Table S4. Comparison of nutrition intake between groups for patients characterized as responders vs. suboptimal responders at 18 months post-surgery; Table S5. Comparison of nutrition intake between outcome groups at 12 months post-surgery; Figure S4. Dietary intake between 12 and 24 months; Figure S5. Proportion of responder and suboptimal responder patients consuming the recommended daily protein; Figure S6. Energy ratio comparisons between outcome groups.
