# Supplementary material for: Longer-Term Weight Loss Outcomes Are Not Primarily Driven by Diet Following Roux-en-Y Gastric Bypass and Sleeve Gastrectomy
Source: Nutrients. 2023 Jul 26;15(15):3323. doi: 10.3390/nu15153323 (PMC10420962; doi:10.3390/nu15153323)
Supplement: Supplementary file 1 [file nutrients-15-03323-s001.zip › nutrients-2452005-supplementary.pdf]

Supplementary Material

Table S1. Study enrollment criteria.

**Inclusion Criteria**	**Method of Assessment**
Male or Female between the ages of 18-65 years, inclusive	Photo Identification
In evaluation for Roux-en-Y Gastric Bypass or Sleeve Gastrectomy as primary procedure	Surgery Department
Planning to be available for two years of follow-up after surgery	Self-report
**Exclusion Criteria**	**Method of Assessment**
Alcohol or substance use disorder in the past year	Structured Interview
Severe psychiatric disorder that may affect the ability of the participant's ability to comply with the protocol	Structured Interview
Tobacco use in the prior year	Self-report
Current medication known to significantly influence gastrointestinal transit time (e.g., opioids, glp-1 agonists, metoclopramide) or is being taken routinely for weight loss (e.g., phentermine, topiramate)	Medical History
Use of any oral or injectable antibiotic in the past month	Medical History
Use of commercially available prebiotic or probiotic in the past month	Medical History
History of significant intestinal disease or disorder that would influence the microbiome (e.g., Crohn's Disease, etc.)	Medical History
History of significant gastrointestinal surgery that would influence the microbiome, not including cholecystectomy or appendectomy	Medical History
Inability to engage in physical activity or dietary monitoring or any medical condition that would put the participant at risk in the study	Self-report
Positive urine drug screen for a non-prescribed medication	Urine drug screen
Pregnancy or breast feeding	Self-report

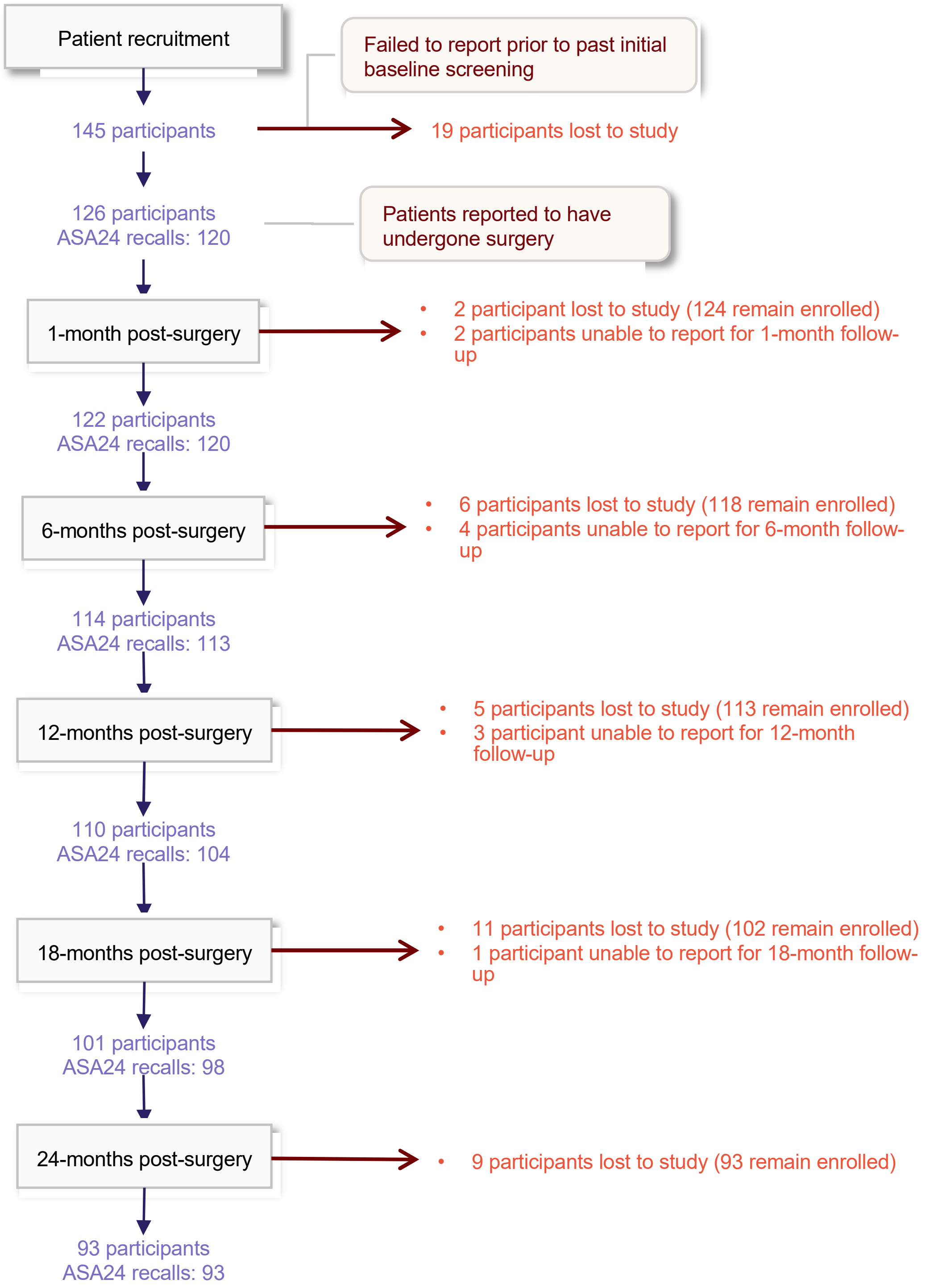

Figure S1. Infographic for participant attrition.

Table S2. Baseline demographics for all participants.

Variable	Total (n = 124)	
Age (years)	42.8 ± 10.2	
Location		
Cleveland	64 (51.6%)	
Fargo	60 (48.4%)	
Sex		
Female	100 (80.6%)	
Male	24 (19.4%)	
Type of Surgery		
Gastric Bypass	87 (70.2%)	
Sleeve Gastrectomy	37 (29.8%)	
Ethnicity		
Black or African American	23 (18.5%)	
Caucasian	92 (74.2%)	
Hispanic or Latino	2 (1.6%)	
Native Hawaiian/Pacific Islander	1 (0.8%)	
More than one race	6 (4.8%)	
Baseline Metrics	RYGB	SG
Weight (kg)	129.5 ± 21.6	136.7 ± 32.0
BMI (kg/m^2)	45.3 ± 5.7	47.5 ± 8.9

Data are reported as mean ± SD for continuous variables and n (%) for categorical variables.

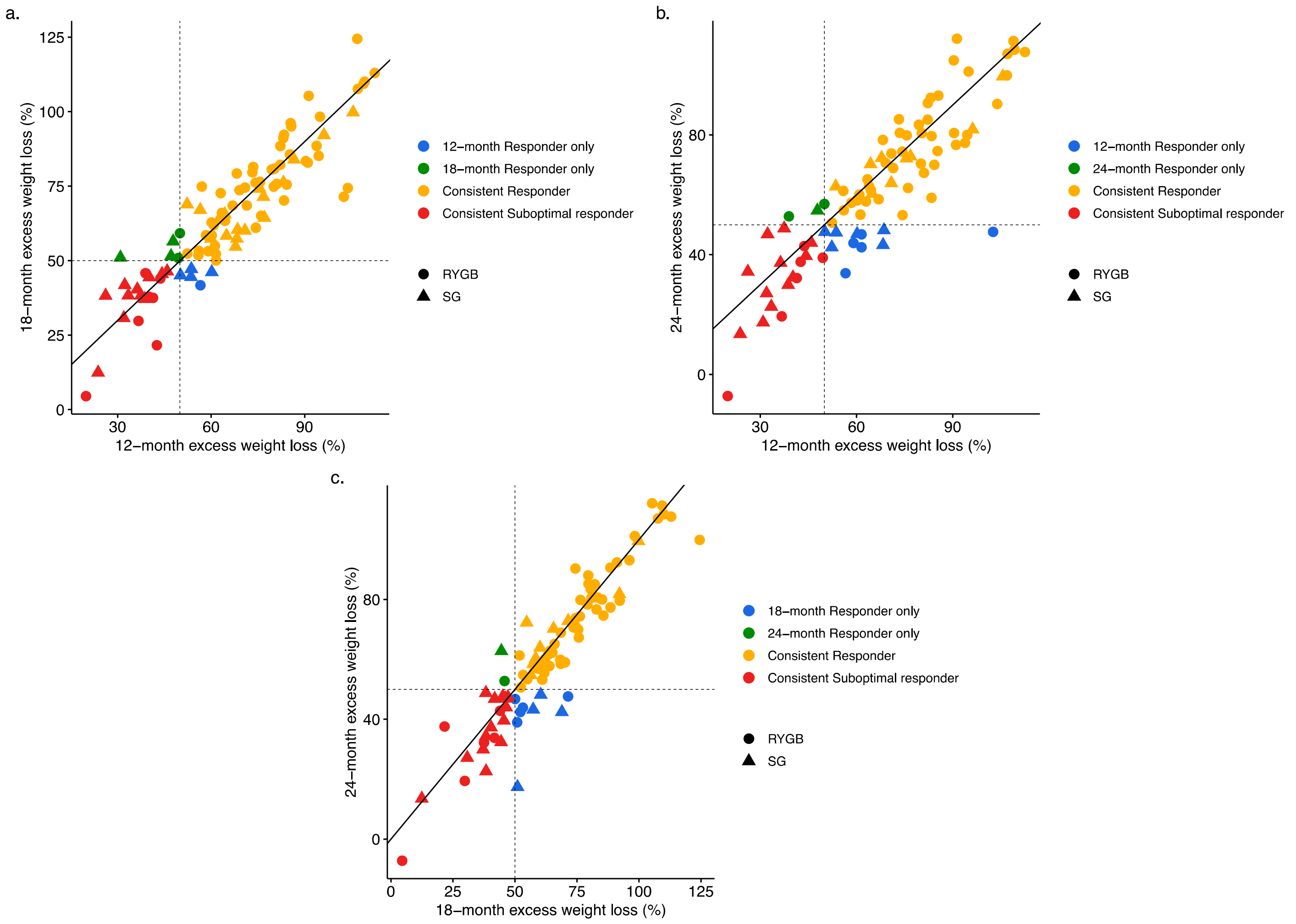

Figure S2. Patient excess weight loss (%) from baseline weight at (a) 12-month v 18-month (Spearman $p < 0.001$; $r = 0.92$), (b) 12-month v 24-month ($p < 0.001$; $r = 0.88$), and (c) 18-month v 24-month ($p < 0.001$; $r = 0.92$). The dashed lines correspond to the 50% EWL response criterium. The solid black line corresponds to the identity line. Points are colored by patient outcome classification consistency and shapes represent patient surgery type.

Table S3. Differences in dietary intake between RYGB and SG patients.

a. Differences in dietary intake between RYGB and SG patients at each timepoint.

	Energy (kcal)			Carbohydrates (g)			Protein (g)			Total fat (g)		
	RYGB	SG	*p*-value	RYGB	SG	*p*-value	RYGB	SG	*p*-value	RYGB	SG	*p*-value
Baseline	1867.34 ± 771.65	1888.68 ± 904.16	0.628	189.66 ± 81.61 (42.5%)	219.93 ± 160.32 (47.8%)	0.993	98.11 ± 41.13 (24.8%)	134.52 ± 127.69 (34%)	0.299	86.81 ± 51.88 (46.6%)	126.02 ± 132.26 (67.1%)	0.231
Postop 1 month	695.34 ± 281.61	804.36 ± 292.1	0.066	60.92 ± 48.25 (35.7%)	65.07 ± 52.81 (32.1%)	0.984	57.74 ± 39.7 (34.6%)	69.05 ± 41.65 (36.2%)	0.034	31.48 ± 36.22 (42.4%)	41.52 ± 41.95 (46.9%)	0.016
Postop 6 month	1047.58 ± 430.56	1140.78 ± 479.64	0.297	94.79 ± 59.89 (35.8%)	96.33 ± 64.76 (33.1%)	0.983	70.01 ± 45.58 (28.2%)	85.01 ± 52.69 (32.1%)	0.046	49.79 ± 42.89 (42.7%)	57.87 ± 45.18 (46.2%)	0.266
Postop 12 month	1216.3 ± 400.05	1230.09 ± 525.16	0.582	116.97 ± 57.96 (37.8%)	100.9 ± 65.83 (30.6%)	0.109	73.3 ± 37.63 (25.2%)	77.12 ± 26.12 (27.1%)	0.219	55.54 ± 34.7 (40.7%)	55.88 ± 25.85 (41.2%)	0.981
Postop 18 months	1331.8 ± 525.44	1216.23 ± 463.51	0.386	129.89 ± 62.07 (38.5%)	107.82 ± 53.51 (34.3%)	0.16	69.53 ± 23.51 (22.3%)	73.03 ± 30.37 (25.1%)	0.609	57.13 ± 25.49 (38.1%)	51.84 ± 21.09 (38.9%)	0.377
Postop 24 months	1240.25 ± 454.72	1268.91 ± 574.29	0.878	120.22 ± 59.02 (41.1%)	114.62 ± 76.56 (33.6%)	0.45	73.88 ± 41.62 (26.9%)	74.44 ± 21.5 (26.8%)	0.409	58.35 ± 42.64 (47.9%)	50.63 ± 22.65 (37.1%)	0.542

Data are reported as mean ± SD. *P*-values were computed by Wilcoxon's rank-sum test. Data in parentheses report the average energy ratio for each macronutrient intake.

b. Differences in dietary intake for RYGB and SG patients relative to baseline intake.

	RYGB				SG			
Timepoint	Calories	Carbohydrates	Protein	Total fat	Calories	Carbohydrates	Protein	Total fat
**BL – 1M**	1.17E-40	1.06E-33	9.53E-11	3.86E-17	1.94E-13	5.85E-12	2.00E-05	7.21E-08
**BL – 6M**	9.64E-22	1.36E-19	8.14E-06	2.37E-08	2.40E-07	4.48E-08	0.001322692	1.49E-05
**BL – 12M**	1.13E-13	1.22E-11	0.000119676	4.43E-06	5.04E-06	1.77E-07	0.000210171	9.98E-06
**BL – 18M**	2.40E-09	4.91E-08	1.32E-05	1.28E-05	5.22E-06	1.29E-06	9.59E-05	5.22E-06
**BL – 24M**	3.17E-12	2.77E-10	0.000217192	3.36E-05	3.36E-05	8.13E-06	0.000217192	8.13E-06

P-values were computed by univariate linear regression and adjusted using the BH procedure.

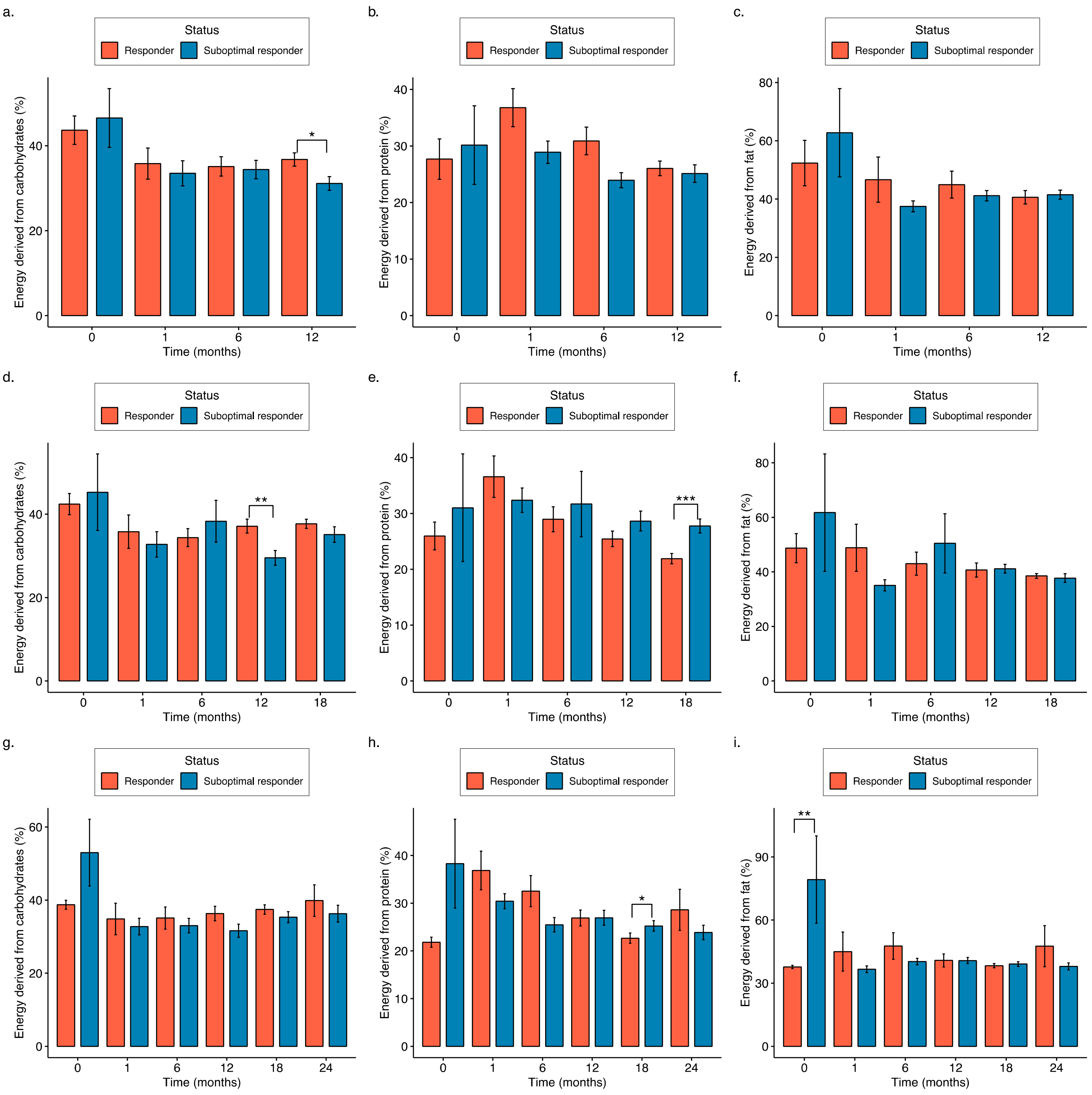

Figure S3. Energy ratio comparisons between outcome groups when classified at 12-months (a-c), 18-months (d-f), and 24-months (g-i) post-surgery. Data are presented as means ± standard errors. Statistical differences are analyzed by Wilcoxon's rank-sum test. * indicates $p < 0.05$, ** indicates $p < 0.01$, and *** indicates $p < 0.001$ responder versus suboptimal responder.

Table S4. Comparison of nutrition intake between groups for patients characterized as responders vs. suboptimal responders at 18 months post-surgery.

		Responder	Suboptimal responder		
		(*n* = 78)	(*n* = 23)	R^2	*p*-value
**Baseline**	**Weight (kg)**	125.68 ± 22.74	145.23 ± 31.02	0.1	**0.003**
	**Energy (kcal)**	1808.77 ± 773.15	1984.93 ± 804.29	0.009	0.402
	**Carbohydrates (g)**	188.42 ± 96.05 (42.4%)	216.43 ± 158.49 (45.3%)	0.011	0.997
	**Protein (g)**	103.8 ± 61.56 (26%)	128.95 ± 138.25 (31%)	0.015	0.144
	**Total fat (g)**	93.02 ± 71.59 (48.7%)	118.03 ± 139.17 (61.7%)	0.013	0.18
**Postop 1 month**	**Weight (kg)**	109.37 ± 18.54	130.85 ± 28.84	0.156	**<0.001**
	**Energy (kcal)**	690.77 ± 277.94	852.43 ± 232.15	0.062	**0.003**
	**Carbohydrates (g)**	61.88 ± 55.06 (35.8%)	69.45 ± 38.19 (32.8%)	0.004	0.107
	**Protein (g)**	60.02 ± 46.67 (36.6%)	67.96 ± 25.15 (32.4%)	0.006	**0.029**$^{\Delta}$
	**Total fat (g)**	35.84 ± 46.42 (48.9%)	33.97 ± 15.5 (35.1%)	<0.001	0.31
**Postop 6 months**	**Weight (kg)**	91.86 ± 16.93	117.64 ± 25.95	0.242	**<0.001**
	**Energy (kcal)**	1024.27 ± 405.75	1288.53 ± 496.14	0.063	**0.021**$^{\Delta}$
	**Carbohydrates (g)**	89.56 ± 57.4 (34.4%)	119.33 ± 69.25 (38.3%)	0.041	0.058$^{\Delta}$
	**Protein (g)**	70.74 ± 46.81 (29%)	94.61 ± 61.59 (31.7%)	0.038	**0.014**
	**Total fat (g)**	48.8 ± 42.99 (43%)	68.95 ± 53.32 (50.5%)	0.033	**0.022**$^{\Delta}$
**Postop 12 months**	**Weight (kg)**	86.05 ± 16.4	116.49 ± 23.99	0.334	**<0.001**
	**Energy (kcal)**	1197.46 ± 424.22	1309.02 ± 501.45	0.012	0.548$^{\Delta}$
	**Carbohydrates (g)**	114.47 ± 62.45 (37.1%)	102.35 ± 59.55 (29.5%)	0.007	0.313$^{\Delta}$
	**Protein (g)**	71.99 ± 36.89 (25.4%)	87.46 ± 26.05 (28.6%)	0.035	**0.002**$^{\Delta}$
	**Total fat (g)**	54.39 ± 34.57 (40.7%)	60.34 ± 27.51 (41.2%)	0.006	0.364$^{\Delta}$
**Postop 18 months**	**Weight (kg)**	86.18 ± 15.96	117.51 ± 21.64	0.368	**<0.001**
	**Energy (kcal)**	1307.61 ± 544.68	1247.25 ± 350.57	0.002	0.922
	**Carbohydrates (g)**	126.52 ± 63.97 (37.7%)	109.43 ± 42.45 (35.1%)	0.014	0.252
	**Protein (g)**	66.52 ± 24.52 (21.9%)	85.02 ± 25.69 (27.8%)	0.09	**0.004**
	**Total fat (g)**	56.07 ± 25.31 (38.5%)	53.11 ± 20.03 (37.7%)	0.003	0.724

Data are reported as mean ± SD. *P*-values were computed by Wilcoxon's rank-sum test. Data in parentheses report the average energy ratio for each macronutrient intake. R^2 was calculated by univariate linear models. Bold *p* values indicate $p < 0.05$ responder versus suboptimal responder. $^{\Delta}$ corresponds with significant comparisons observed by Lim et al. (2020).

Table S5. Comparison of nutrition intake between outcome groups at 12 months post-surgery. The sample size was limited to those patients who participated for the entirety of the study.

		**Responder**	**Suboptimal responder**		
		**(*n* = 69)**	**(*n* = 21)**	**R^2**	***p*-value**
**Baseline**	**Weight (kg)**	126.01 ± 21.13	146.67 ± 31.57	0.127	**0.004**
	**Energy (kcal)**	1809.5 ± 684.94	1887.69 ± 783.98	0.002	0.805
	**Carbohydrates (g)**	191.25 ± 117.75 (41.7%)	203.48 ± 107.7 (47.9%)	0.002	0.589
	**Protein (g)**	104.8 ± 88.73 (25.5%)	118.73 ± 75.59 (32.6%)	0.005	0.209
	**Total fat (g)**	90.69 ± 90.82 (46.6%)	111.26 ± 76.99 (66.8%)	0.01	0.085
**Postop 1 month**	**Weight (kg)**	110.27 ± 18.12	130.76 ± 29.13	0.173	**0.001**
	**Energy (kcal)**	669.9 ± 238.59	932.39 ± 310.73	0.161	**<0.001**
	**Carbohydrates (g)**	55.97 ± 45.43 (33.9%)	82.23 ± 47.99 (34.7%)	0.056	**0.007**
	**Protein (g)**	59.33 ± 41.28 (36.8%)	64.42 ± 23.15 (28.9%)	0.003	0.170$^{\Delta}$
	**Total fat (g)**	31.49 ± 39.02 (44.5%)	38.42 ± 17.98 (36.4%)	0.007	**0.02**
**Postop 6 months**	**Weight (kg)**	91.16 ± 14.14	120.43 ± 26.51	0.331	**<0.001**
	**Energy (kcal)**	1009.64 ± 429.35	1316.72 ± 497.32	0.077	**0.010**$^{\Delta}$
	**Carbohydrates (g)**	88.61 ± 64 (34.7%)	112.82 ± 59.37 (34%)	0.025	**0.037**$^{\Delta}$
	**Protein (g)**	77.27 ± 58.3 (32.2%)	76.97 ± 30.74 (23.7%)	<0.001	0.391
	**Total fat (g)**	51.92 ± 52.6 (46.5%)	61.28 ± 26.7 (41.7%)	0.007	**0.016**$^{\Delta}$
**Postop 12 months**	**Weight (kg)**	85.28 ± 14.28	118.58 ± 23.99	0.413	**<0.001**
	**Energy (kcal)**	1143.57 ± 426.84	1396.66 ± 472.81	0.058	0.064$^{\Delta}$
	**Carbohydrates (g)**	107.97 ± 65.21 (36.1%)	112.72 ± 59.42 (30.9%)	0.001	0.744$^{\Delta}$
	**Protein (g)**	73.28 ± 37.92 (27.3%)	84.63 ± 26.53 (25.6%)	0.018	**0.021**$^{\Delta}$
	**Total fat (g)**	52.06 ± 35.84 (40.7%)	64.45 ± 26.74 (41.2%)	0.024	**0.035**$^{\Delta}$

Data are reported as mean ± SD. *P*-values were computed by Wilcoxon's rank-sum test. Data in parentheses report the average energy ratio for each macronutrient intake. R^2 was calculated by univariate linear models. Bold *p* values indicate $p < 0.05$ responder versus suboptimal responder. $^{\Delta}$ corresponds with significant comparisons observed by Lim et al. (2020).

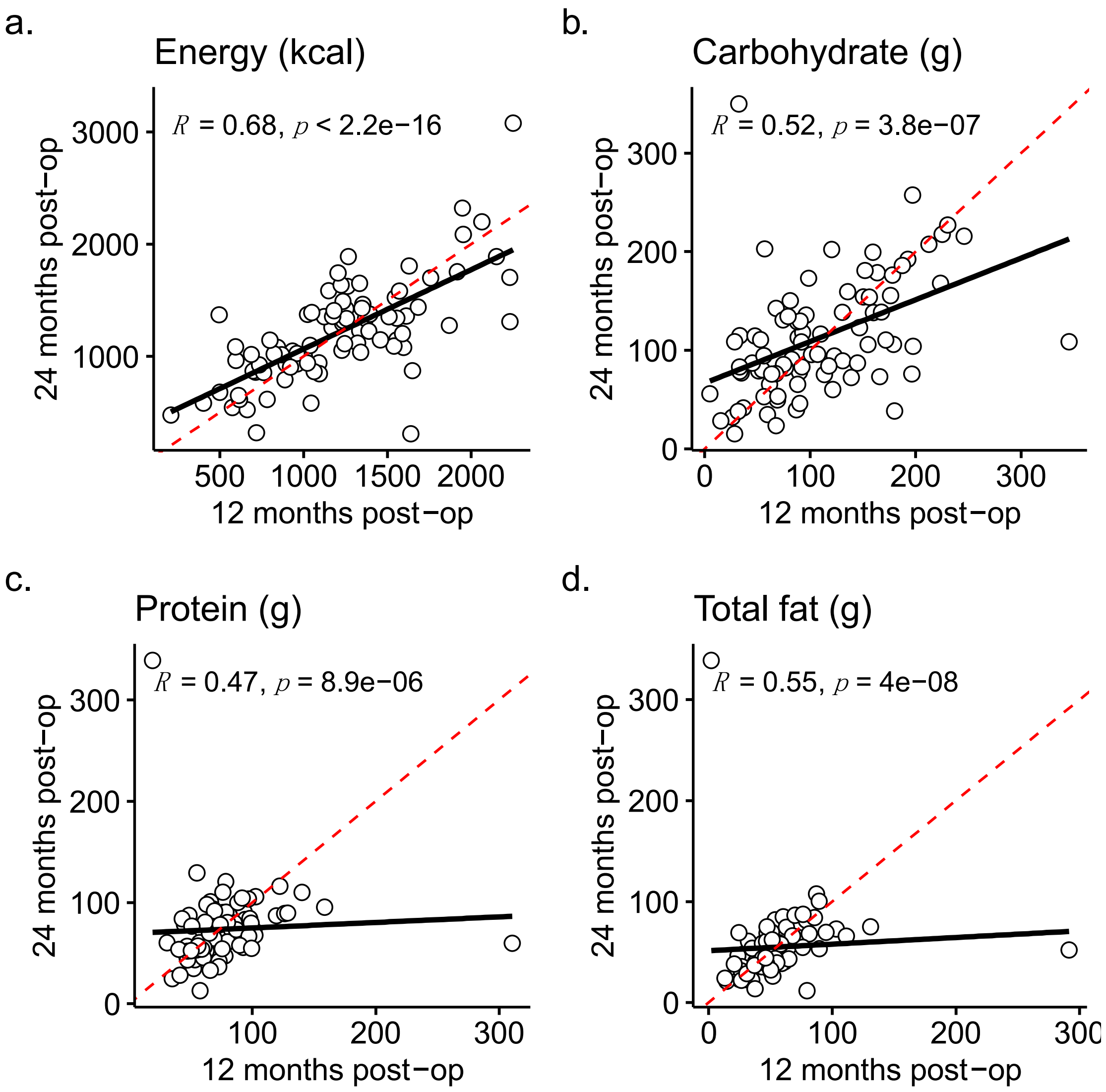

Figure S4. Dietary intake between 12 and 24 months (a) calories, (b) carbohydrates, (c) protein, (d) total fat. The dashed red line corresponds to the identity line. Correlation coefficients (*r*) and *p*-values were calculated using Spearman's rank correlation.

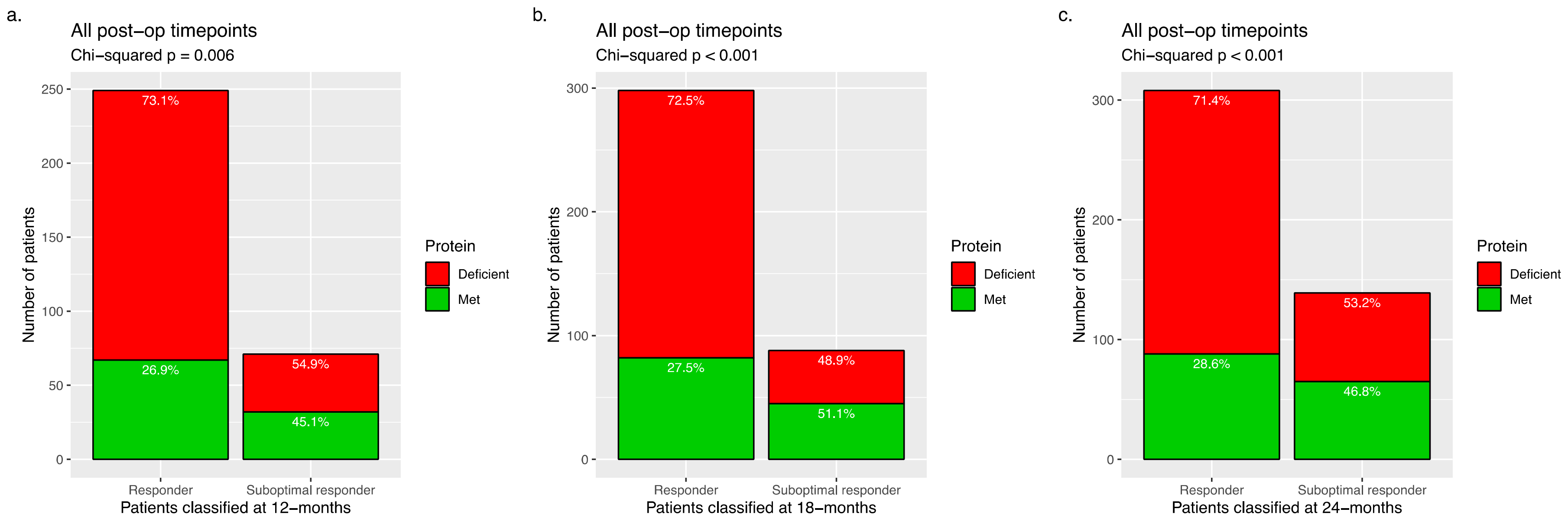

Figure S5. Proportion of responder and suboptimal responder patients consuming the recommended daily 1.1-1.5 g protein per kg ideal body weight when weight loss outcomes were classified at (a) 12-months, (b) 18-months, and (c) 24-months after surgery. Proportions were calculated by patients meeting the minimum recommended intake at each post-surgical assessment up to the month of weight loss outcome classifications.

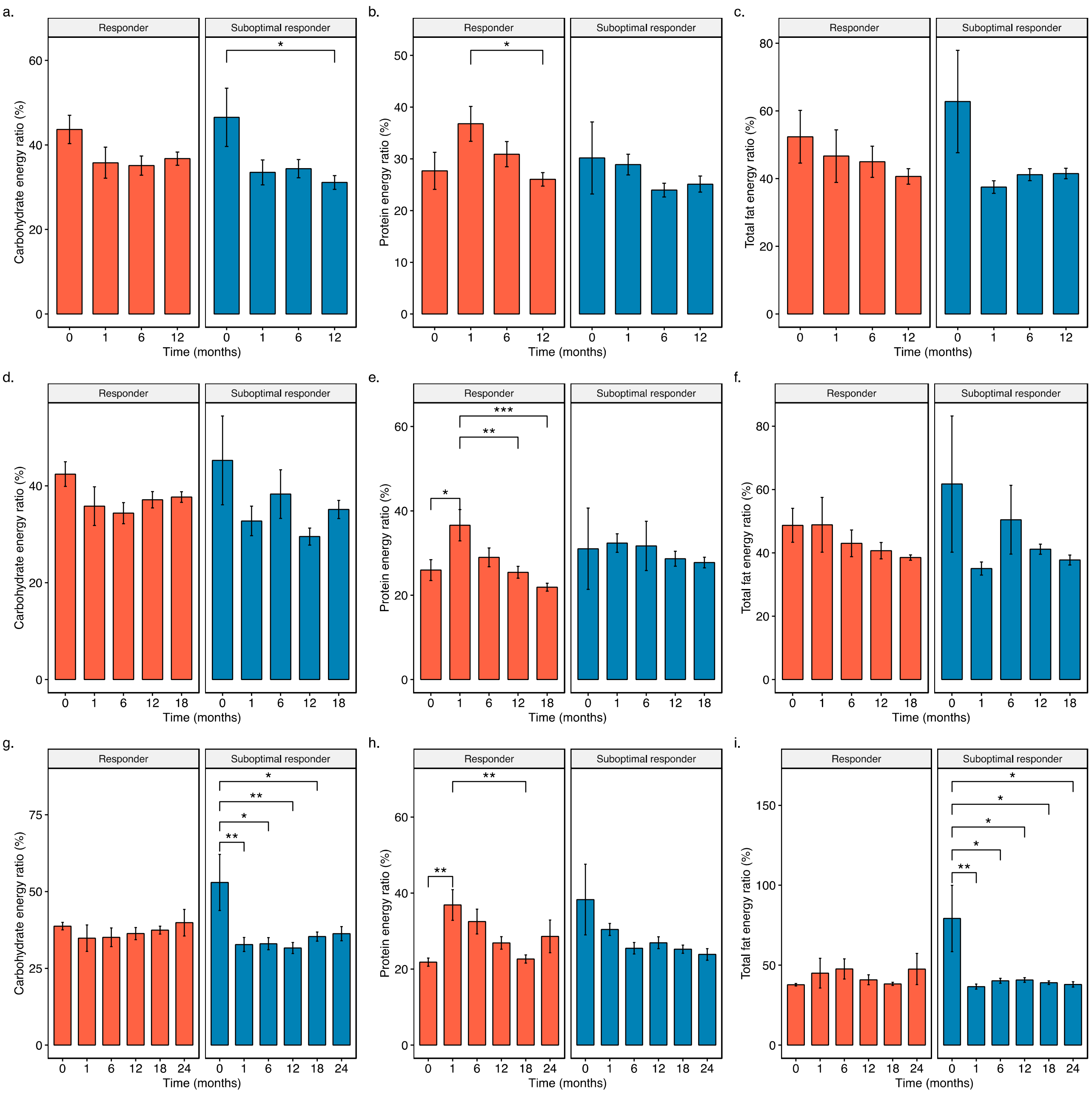

Figure S6. Energy ratio comparisons between outcome groups when classified at 12-months (a-c), 18-months (d-f), and 24-months (g-i) post-surgery. Data are presented as means ± standard errors. Statistical differences are analyzed by Tukey's HSD test. * indicates $p < 0.05$, ** indicates $p < 0.01$, and *** indicates $p < 0.001$ between time points.
